# Exploring Disease-Specific Waitlist Outcomes in Simultaneous Liver-Kidney Transplantation

**DOI:** 10.3389/ti.2026.16153

**Published:** 2026-04-13

**Authors:** Rikako Oki, Luckshi Rajendran, Dean Yonghoon Kim, Atsushi Yoshida, Marwan Abouljoud, Shunji Nagai

**Affiliations:** 1 Transplant Institute, Henry Ford Hospital, Detroit, MI, United States; 2 Division of Transplant and Hepatobiliary Surgery Henry Ford Hospital, Detroit, MI, United States

**Keywords:** allocation policy change, liver etiology, metabolic dysfunction-associated steatohepatitis, simultaneous liver-kidney transplantation, waitlist outcomes

## Abstract

The current allocation system does not account for liver etiology in simultaneous liver-kidney transplantation (SLKT). This study aims to assess differences in waitlist outcomes among major liver disease groups (alcohol-related liver disease [ALD], metabolic dysfunction-associated steatohepatitis [MASH], hepatitis C virus infection, and biliary diseases) in SLKT using Organ Procurement and Transplantation Network (OPTN) registry. In total, 4,846 adult SLKT candidates listed between January 2018 and March 2024 were enrolled. Patients with MASH had worse waitlist 1-year mortality compared to ALD adjusted for patient characteristics at listing (HR 1.300, 95% CI 1.059–1.597, p = 0.012), whereas the 1-year SLKT probability was comparable. When patients were categorized by MELD score at listing (6–20, 21–29, and ≥30), patients with MASH had significantly higher 1-year waitlist mortality compared to those with ALD in the middle MELD score group (HR 1.365, 95% CI 1.008–1.834, p = 0.044). Prior to the allocation policy change in 2020, patients with MASH experienced higher waitlist mortality compared to ALD, however, this disparity was not observed following the policy change. Waitlist outcomes varied significantly depending on the etiology in SLKT. The revised 2020 allocation policy may be temporally associated with changes in mortality disparities across different liver etiologies.

## Graphical Abstract

**Figure:**
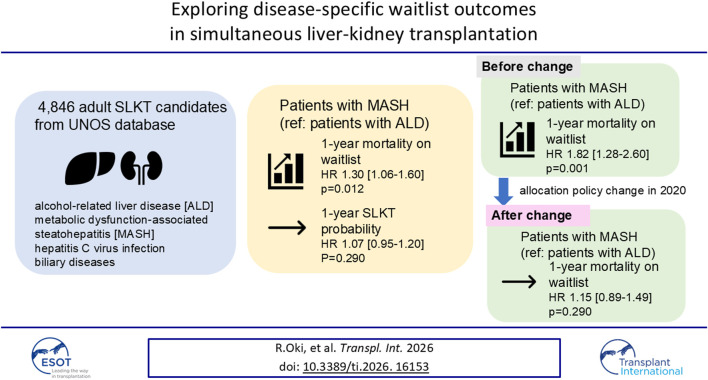

## Introduction

Simultaneous liver and kidney transplantation (SLKT) is an appropriate option for those suffering from end-stage liver disease and critical renal insufficiency. Following the introduction of the Model for End-Stage Liver Disease (MELD) scoring system in 2002, the number of SLKT has increased because serum creatinine substantially impacts MELD score prioritization [1]. Due to concern about the potentially disproportionate use of renal allografts in SLKT, standardized medical criteria were established in 2017 to promote equitable allocation between kidney and liver transplant candidates [2, 3]. We previously reported that following the 2017 policy change, the number of SLKT declined despite increased registrations, while LT (liver transplantation) alone increased and were associated with worse 1-year graft survival (GS), highlighting the need for ongoing evaluation of transplant policy [4].

As the MELD score does not incorporate the etiology of liver disease, the current allocation system does not account for liver etiology. We previously evaluated disease-specific differences in waitlist outcomes among LT alone candidates with different underlying etiologies, including alcohol-related liver disease (ALD), nonalcoholic steatohepatitis (NASH), hepatitis C virus infection (HCV), primary biliary cirrhosis (PBC), and primary sclerosing cholangitis [5]. Patients with NASH had a significantly higher risk of 1-year waitlist mortality than those with ALD at comparable MELD scores [5]. The recent retrospective studies also showed that patients with metabolic dysfunction-associated steatohepatitis (MASH) had higher LT waitlist mortality [6, 7]. An Italian registry study showed that patients with MASH were associated with higher LT waitlist mortality, while these candidates demonstrated the greatest 5-year LT survival benefit at the same MELD score [6]. However, few studies have evaluated potential differences in disparities in waitlist outcomes among major liver disease etiologies in the context of SLKT.

This study aims to assess differences in waitlist outcomes among major liver disease groups, including ALD, HCV, MASH and biliary diseases by using Organ Procurement and Transplantation Network (OPTN) dataset in the contemporary SLKT policy era established in 2017. On February 4, 2020, the liver allocation system in the United States underwent a major revision, replacing donation service areas with acuity circles as the geographic basis for organ distribution, and shifting from a predominantly local allocation approach to a broader sharing model [8]. Therefore, we also examined whether disease-specific waitlist outcomes changed before and after the allocation system change in 2020.

## Materials and Methods

### Patients and Data Collection

This was a retrospective cohort study using OPTN database. We included all adult (age at listing >18 years) SLKT candidates who were listed between January 2018 and March 2024 with last follow-up as of March 2025, and whose primary liver disease etiologies were ALD, MASH, HCV, and biliary diseases. Biliary diseases included primary biliary cholangitis, primary sclerosing cholangitis, and other biliary diseases. Following the consensus policy enacted by UNOS in 2017, candidates for SLKT must meet specific criteria, including having chronic kidney disease (CKD), sustained acute kidney injury (AKI), or a metabolic disease, as diagnosed by a nephrologist [9]. For CKD, eligibility requires an estimated glomerular filtration rate (eGFR) ≤60 mL/min for at least 90 days, an eGFR ≤30 mL/min at the time of listing, or ongoing chronic dialysis [9]. For sustained AKI, candidates must have received dialysis for a minimum of 6 weeks or have had documented eGFR values ≤25 mL/min during that period [9]. Patient characteristics at listing included age, sex, race, liver disease etiology, frailty level, body mass index (BMI), dialysis, creatinine (Cr), diabetes, ascites, encephalopathy, life support use, serum sodium level, total bilirubin, international normalized ratio (INR) and MELD score. Race was classified into five categories; White, Black, Hispanic, Asian, and Others. The frailty level was based on the Karnofsky Performance Status, with scores of 80 or higher classified as normal, scores of 50–70 classified as mild frailty, and scores of 10–40 classified as severe frailty [10]. Exclusion criteria included the following: (1) LT combined with thoracic organ(s), pancreas, and/or intestine, (2) patients with MELD exception for HCC and other reasons (non-HCC condition), (3) patients whose liver etiology was not classified into ALD, MASH, HCV, and biliary diseases, (4) patients with overlapping liver disease etiologies (e.g., HCV and ALD).

This study used the OPTN dataset provided by the United Network for Organ Sharing (UNOS), in which all individually identifiable information is encrypted. Henry Ford Institutional Review Board (IRB) exempted this study from IRB approval. All methods of research procedures were performed in accordance with the Declaration of Helsinki.

### Waitlist Outcomes

The waitlist outcomes included mortality and SLKT. Removal from the waitlist due to clinical deterioration (too sick for transplantation) was included in mortality. Mortality, SLKT, LT alone (not undergoing KT (kidney transplantation) due to recovery of kidney function or other reasons), recovery of liver function (too well for transplantation), and removal from the waitlist for other reasons were considered competing risk events. Differences in waitlist outcomes between liver etiologies were evaluated. ALD was selected as the reference etiology for the following reasons: (1) it represents one of the major underlying causes of SLKT, (2) it was used as the reference group in our prior study of LT waitlist outcomes [5], and (3) it has been shown to have a lower risk of mortality on the LT waitlist compared with other liver diseases [11].

First, 90-day and 1-year waitlist outcomes were compared across the four liver disease etiologies. Then, patients were stratified into three categories based on their MELD score at listing (6–20, 21–29, and ≥30), and 90-day and 1-year waitlist outcomes were subsequently compared across the disease etiologies.

To assess whether waitlist outcomes among liver disease etiologies differed before and after the February 4, 2020 allocation policy change, we defined two periods: Period 1 (listed from January 1, 2018 to February 3, 2020) and Period 2 (listed from February 4, 2020 to March 31, 2024). Patients were assigned to Period 1 if their listing initiation date was before February 4, 2020, and to Period 2 if their initiation date was on or after February 4, 2020. For those listed in Period 1 whose removal date extended beyond February 3, 2020, follow-up was administratively censored at February 3, 2020. Waitlist outcomes were then compared across liver etiologies in each period separately.

#### Post-Transplantation Outcomes

Among patients who underwent SLKT, we also examined the association between etiology and patient death within 1 year as well as patient death within 3 years. We further assessed whether cause-specific mortality varied across etiologies.

### Statistical Analyses

All statistical analyses were conducted using the following software (SPSS®, Version <28.0.1>; IBM Corp., Armonk, NY, USA), EZR (Saitama Medical Center, Jichi Medical University, Saitama, Japan, Version <1.70>), and a graphical user interface for R (R Foundation for Statistical Computing, Vienna, Austria). Continuous data were expressed as mean ± standard deviation or median (interquartile range). Student’s t-tests or Mann–Whitney U-tests were used to compare continuous variables. The chi-square test or Fisher’s exact test was used to compare the categorical variables. To assess differences in 90-day and 1-year waitlist outcomes, the cumulative incidence of competing events was compared using Gray’s test, and multivariable Fine-Gray proportional hazards regression was performed to identify risk factors. Among patients who underwent SLKT, we evaluated the association between etiology and cause-specific mortality using multivariable Fine–Gray proportional hazards regression. Multivariable Cox regression analysis was performed to examine the association between liver etiology and patient death among patients who received SLKT. The risks were adjusted for patient characteristics at listing, including age [12], ascites [13], BMI [12], diabetes [12], encephalopathy [14], frailty [15], dialysis [13], life support measures [13], sex [16], MELD score [16], and race [17], which have been reported to be factors associated with waitlist outcomes for LT. *P-values* less than 0.05 were considered as significant.

## Results

### Characteristics of Study Participants

In total, 8,026 adult SLKT candidates were listed during the study period. Excluding 110 candidates listed with other organs at the same time and 328 patients with MELD exception, a total of 7,588 patients were identified. Among them, only those with ALD (n = 1,751), HCV (n = 628), MASH (n = 2,183), or biliary diseases (n = 285) were included in the study cohort (Total n = 4,846). Table 1 presents the baseline characteristics of all patients. The median patient age was 60.0 years and females accounted for 43.5%. The median MELD score at listing was 24, and approximately half of the recipients were already on dialysis. Patient backgrounds differed significantly across all etiologies of liver dysfunction. Patients with MASH had the highest age, BMI, and prevalence of diabetes mellitus. In patients with ALD, the rate of dialysis and moderate to severe hepatic encephalopathy was higher than in other groups, and their MELD scores were the highest among all etiologies.

**TABLE 1:** Background characteristics of patients on waitlist.

Variables	All (N = 4,846)	ALD (N = 1,751)	HCV (N = 628)	MASH (N = 2,183)	Biliary disease (N = 285)	P value
Age (y.o.)	60.0 [53.0, 66.0]	55.0 [45.0, 62.0]	61.0 [57.0, 65.0]	63.0 [57.0, 67.0]	61.0 [52.0, 66.0]	<0.001
Female (%)	2,107 (43.5)	521 (29.8)	198 (31.5)	1,220 (55.9)	169 (59.3)	<0.001
BMI	28.4 [24.7, 33.1]	26.9 [23.4, 30.9]	27.1 [23.9, 31.1]	30.8 [26.9, 35.4]	25.8 [22.6, 29.9]	<0.001
Diabetes (%)	2,527 (52.2)	439 (25.1)	371 (59.1)	1,609 (73.8)	108 (36.9)	<0.001
Race (%)	​	​	​	​	​	<0.001
White	3,106 (64.1)	1,201 (68.6)	266 (42.4)	1,474 (67.5)	165 (57.9)	​
Black	418 (8.6)	104 (5.9)	174 (27.7)	74 (3.4)	66 (23.2)	​
Hispanic	1,062 (21.9)	362 (20.7)	154 (24.5)	509 (23.3)	38 (13.3)	​
Asian	164 (3.4)	48 (2.7)	26 (4.1)	83 (3.8)	7 (2.5)	​
Others	96 (2.0)	36 (2.1)	8 (1.3)	43 (2.0)	9 (3.2)	​
Albumin (g/dL)	3.30 [2.80, 3.70]	3.30 [2.80, 3.70]	3.40 [2.90, 3.90]	3.20 [2.80, 3.70]	3.20 [2.70, 3.70]	<0.001
Bilirubin (mg/dL)	1.70 [0.90, 3.80]	2.20 [1.10, 6.15]	0.90 [0.50, 1.90]	1.60 [0.90, 3.10]	2.10 [0.90, 7.70]	<0.001
Creatinine (mg/dL)	2.94 [1.95, 4.61]	3.11 [2.04, 4.70]	3.92 [2.33, 6.12]	2.68 [1.85, 4.11]	2.90 [1.96, 4.42]	<0.001
INR	1.40 [1.20, 1.76]	1.50 [1.20, 1.90]	1.20 [1.10, 1.50]	1.40 [1.20, 1.70]	1.30 [1.10, 1.60]	<0.001
Sodium	136 [133, 139]	136 [133, 138]	138 [135, 140]	137 [134, 139]	137 [134, 140]	<0.001
MELD score	24 [21, 31]	27 [22, 34]	22 [20, 26]	23 [20, 29]	23 [20, 31]	<0.001
Dialysis (%)	2,389 (49.3)	1,039 (59.3)	359 (57.3)	872 (39.9)	119 (41.8)	<0.001
Life support measure (%)	345 (7.1)	166 (9.5)	39 (6.2)	122 (5.6)	18 (6.3)	<0.001
Ascites (%)	2,165 (44.7)	902 (51.5)	208 (33.1)	1,027 (47.0)	82 (28.2)	<0.001
Encephalopathy	​	​	​	​	​	<0.001
None/mild (%)	4,320 (89.1)	1,523 (87.0)	583 (92.8)	1945 (89.1)	270 (94.7)	​
Moderate/Severe (%)	526 (10.9)	228 (13.0)	45 (7.2)	238 (10.9)	15 (5.3)	​
Frailty	​	​	​	​	​	<0.001
Normal (%)	624 (13.1)	181 (10.5)	99 (15.9)	301 (14.1)	43 (15.4)	​
Mild (%)	2,248 (47.3)	722 (42.1)	321 (51.6)	1,065 (49.9)	140 (50.0)	​
Severe (%)	1883 (39.6)	814 (47.4)	202 (32.5)	770 (36.0)	97 (34.6)	​

ALD, alcohol-related liver disease; HCV, hepatitis C virus infection; MASH, metabolic dysfunction-associated steatohepatitis; BMI, body mass index; INR, international normalized ratio; MELD, Model for End-Stage Liver Disease.

### Comparison of Waitlist Outcomes Among Liver Disease Etiologies

**FIGURE 1:**
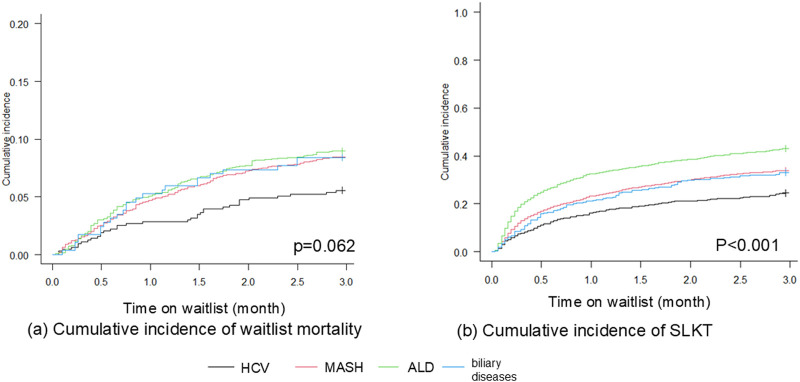
Cumulative incidence curves showing 90-day **(a)** waitlist mortality and **(b)** SLKT probability, among HCV, MASH, ALD, and biliary diseases (Gray test). **(a)** There were no significant differences among liver etiologies for 90-day mortality (p = 0.062). **(b)** Patients with ALD demonstrated the highest 90-day SLKT probability (p < 0.001). ALD, alcohol-related liver disease; HCV, hepatitis C virus infection; MASH, metabolic dysfunction-associated steatohepatitis.

**FIGURE 2:**
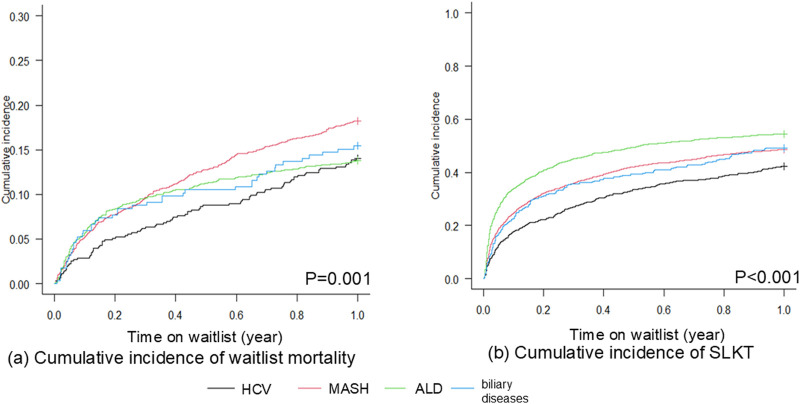
Cumulative incidence curves showing 1-year **(a)** waitlist mortality and **(b)** SLKT probability among HCV, MASH, ALD, and biliary diseases (Gray test). **(a)** Patients with MASH demonstrated the highest 1-year waitlist mortality (p = 0.001). **(b)** Patients with ALD demonstrated the highest 1-year SLKT probability (p < 0.001). ALD, alcohol-related liver disease; HCV, hepatitis C virus infection; MASH, metabolic dysfunction-associated steatohepatitis.

Figure 1 shows the unadjusted cumulative incidence of waitlist outcomes among patients with the four liver disease etiologies. There was no difference in 90-day waitlist mortality according to liver etiology (p = 0.062). ALD patients demonstrated the highest 90-day SLKT probability (p < 0.001). In contrast, patients with MASH experienced significantly higher 1-year waitlist mortality (p < 0.001), whereas patients with ALD had the highest 1-year SLKT probability (p < 0.001, Figure 2). We next evaluated the probability of LT alone (not undergoing KT due to recovery of kidney function or other reasons). ALD patients demonstrated the highest 90-day and 1-year LT alone probability (p < 0.001, p = 0.001, respectively). The 90-day cumulative incidence was 4.6% (95% CI 3.7–5.6) in patients with ALD, 1.8% (0.9–3.0) in HCV, 2.6% (2.0–3.3) in MASH, and 2.1% (0.9–4.3) in biliary disease. The 1-year cumulative incidence was 6.0% (95% CI 5.0–7.2) in patients with ALD, 2.7% (1.6–4.2) in HCV, 4.1% (3.3–5.0) in MASH, and 3.2% (1.6–5.7) in biliary disease (Supplementary Figure S1).

### Adjusted Risks for Waitlist Outcomes in Each Liver Disease Etiology vs. ALD

Adjusted hazards of 90-day and 1-year waitlist mortality and SLKT probability in HCV, MASH and biliary diseases were estimated and compared to the ALD group. No significant difference in 90-day waitlist mortality or SLKT probability was observed among the liver disease etiologies (Table 2). The overall risk of 1-year waitlist mortality was significantly higher in MASH compared to ALD (HR 1.300, 95% CI 1.059–1.597, p = 0.012), whereas the 1-year SLKT probability was comparable between MASH and ALD (Table 3). Regarding the probability of LT alone (not undergoing KT due to recovery of kidney function or other reasons), there was no significant difference between the liver etiologies (Supplementary Table S1).

**TABLE 2:** Multivariable Fine-Gray proportional hazards regression for 90-day waitlist outcomes.

Variables	Death		SLKT	
	HR (95% CI)	*p*-value	HR (95% CI)	*p*-value
Age	1.018 (1.006–1.030)	0.003	1.002 (0.997–1.008)	0.440
Female	1.239 (1.003–1.529)	0.047	0.814 (0.730–0.907)	<0.001
BMI	0.992 (0.974–1.010)	0.360	0.994 (0.986–1.003)	0.200
Diabetes	1.155 (0.907–1.472)	0.240	1.032 (0.916–1.162)	0.610
Etiology of liver disease (ref; ALD)				
HCV	0.949 (0.620–1.453)	0.810	0.887 (0.733–1.073)	0.220
MASH	1.167 (0.882–1.544)	0.280	1.080 (0.963–1.275)	0.150
Biliary disease	1.201 (0.763–1.890)	0.430	0.967 (0.761–1.228)	0.780
Race (ref: White)				
Black	1.065 (0.728–1.559)	0.740	0.821 (0.656–1.029)	0.086
Hispanic	1.028 (0.801–1.320)	0.830	0.726 (0.634–0.831)	<0.001
Asian	0.491 (0.217–1.112)	0.088	0.788 (0.579–1.072)	0.130
Others	0.581 (0.243–1.386)	0.220	0.912 (0.648–1.284)	0.600
MELD score	1.103 (1.086–1.120)	<0.001	1.100 (1.090–1.110)	<0.001
Dialysis	1.138 (0.908–1.427)	0.260	0.929 (0.829–1.039)	0.200
Life support measures	1.070 (0.781–1.463)	0.680	0.774 (0.623–0.962)	0.021
Ascites	1.182 (0.954–1.465)	0.130	1.136 (1.020–1.265)	0.020
Encephalopathy (ref; none or mild)				
Severe	1.037 (0.784–1.372)	0.800	1.044 (0.883–1.235)	0.610
Frailty (ref; normal)				
Severe	2.257 (1.358–3.750)	0.002	1.627 (1.333–1.985)	<0.001

ALD, alcohol-related liver disease; HCV, hepatitis C virus infection; MASH, metabolic dysfunction-associated steatohepatitis; BMI, body mass index; MELD, Model for End-Stage Liver Disease; SLKT, simultaneous liver-kidney transplantation.

**TABLE 3:** Multivariable Fine-Gray proportional hazards regression for 1-year waitlist outcomes.

Variables	Death		SLKT	
	HR (95% CI)	*p*-value	HR (95% CI)	*p*-value
Age	1.023 (1.014–1.032)	<0.001	1.002 (0.997–1.007)	0.480
Female	1.170 (1.005–1.362)	0.043	0.835 (0.761–0.915)	<0.001
BMI	0.984 (0.971–0.997)	0.017	1.001 (0.993–1.008)	0.840
Diabetes	1.062 (0.895–1.259)	0.490	1.033 (0.936–1.141)	0.510
Etiology of liver disease (ref; ALD)				
HCV	1.093 (0.829–1.442)	0.530	0.953 (0.819–1.108)	0.530
MASH	1.300 (1.059–1.597)	0.012	1.066 (0.947–1.200)	0.290
Biliary disease	1.200 (0.857–1.680)	0.290	1.040 (0.856–1.269)	0.680
Race (ref: White)				
Black	0.923 (0.688–1.239)	0.590	0.864 (0.724–1.031)	0.110
Hispanic	1.020 (0.853–1.220)	0.830	0.795 (0.711–0.890)	<0.001
Asian	0.797 (0.511–1.245)	0.320	0.837 (0.654–1.072)	0.160
Others	0.642 (0.341–1.212)	0.170	1.088 (0.827–1.430)	0.550
MELD score	1.042 (1.030–1.055)	<0.001	1.069 (1.061–1.077)	<0.001
Dialysis	1.076 (0.916–1.263)	0.370	0.903 (0.819–0.994)	0.037
Life support measures	1.112 (0.850–1.456)	0.440	0.827 (0.681–1.006)	0.057
Ascites	1.173 (1.005–1.369)	0.043	1.102 (1.006–1.208)	0.037
Encephalopathy (ref; none or mild)				
Severe	1.040 (0.829–1.307)	0.730	1.043 (0.895–1.215)	0.590
Frailty (ref; normal)				
Severe	1.250 (0.956–1.635)	0.100	1.465 (1.258–1.705)	<0.001

ALD, alcohol-related liver disease; HCV, hepatitis C virus infection; MASH, metabolic dysfunction-associated steatohepatitis; BMI, body mass index; MELD, Model for End-Stage Liver Disease; SLKT, simultaneous liver-kidney transplantation.

### Adjusted Risks for Waitlist mortality in Each Liver Disease Etiology vs. ALD According to MELD Score Category at Listing

When evaluating outcomes according to MELD score categories at listing, 90-day waitlist mortality did not significantly differ across liver disease etiologies in any MELD score group. In the higher MELD score subgroup, patients with MASH demonstrated a higher 90-day SLKT probability compared to those with ALD. (HR 1.239, 95% CI 1.038–1.478, p = 0.018, Table 4).

**TABLE 4:** Multivariable Fine-Gray proportional hazards regression for 90-day waitlist outcomes categorized by initial MELD score. Adjusted for baseline characteristics at listing, including age, ascites, BMI, diabetes, encephalopathy, dialysis, life support measures, frailty, sex and race.

Variables	MELD 6–20		MELD 21–29		MELD>30	
	HR (95% CI)	*p*-value	HR (95% CI)	*p*-value	HR (95% CI)	*p*-value
Etiology of liver disease (ref; ALD)						
(a) Death						
HCV	2.293 (0.663–7.925)	0.190	0.697 (0.347–1.399)	0.310	1.260 (0.707–2.241)	0.430
MASH	0.998 (0.323–3.081)	1.000	1.393 (0.867–2.238)	0.170	1.041 (0.726–1.493)	0.830
Biliary disease	Not calculable	​	1.391 (0.611–3.167)	0.430	1.277 (0.748–2.181)	0.370
(b) SLKT						
HCV	1.437 (0.741–2.787)	0.280	0.809 (0.623–1.049)	0.110	0.934 (0.696–1.252)	0.650
MASH	1.717 (0.959–3.076)	0.069	0.934 (0.768–1.135)	0.490	1.239 (1.038–1.478)	0.018
Biliary disease	1.571 (0.687–3.594)	0.280	0.840 (0.589–1.198)	0.340	0.956 (0.709–1.287)	0.760

ALD, alcohol-related liver disease; HCV, hepatitis C virus infection; MASH, metabolic dysfunction-associated steatohepatitis; BMI, body mass index; MELD, Model for End-Stage Liver Disease; SLKT, simultaneous liver-kidney transplantation.

In contrast, the 1-year waitlist mortality in the middle MELD score subgroup was significantly higher (HR 1.365, 95% CI 1.009–1.834, p = 0.044), although the 1-year SLKT probability did not differ across the etiologies (Table 5). In the higher MELD score subgroup, patients with MASH demonstrated a higher 1-year SLKT probability compared to those with ALD. (HR 1.188, 95% CI 1.001–1.410, p = 0.048).

**TABLE 5:** Multivariable Fine-Gray proportional hazards regression for 1-year waitlist outcomes categorized by initial MELD score at listing. Adjusted for baseline characteristics at listing, including age, ascites, BMI, diabetes, encephalopathy, dialysis, dialysis duration, life support measures, frailty, sex and race.

Variables	MELD 6–20		MELD 21–29		MELD>30	
	HR (95% CI)	*p*-value	HR (95% CI)	*p*-value	HR (95% CI)	*p*-value
Etiology of liver disease (ref; ALD)						
(a) Death						
HCV	1.916 (1.018–3.606)	0.044	0.881 (0.601–1.292)	0.520	1.530 (0.928–2.524)	0.095
MASH	1.667 (0.962–2.891)	0.069	1.365 (1.008–1.834)	0.044	1.121 (0.797–1.576)	0.510
Biliary disease	0.722 (0.253–2.062)	0.540	1.386 (0.854–2.250)	0.190	1.203 (0.712–2.034)	0.490
(b) SLKT						
HCV	0.883 (0.595–1.310)	0.540	0.999 (0.817–1.222)	0.990	0.934 (0.708–1.233)	0.630
MASH	1.115 (0.820–1.515)	0.490	0.989 (0.840–1.164)	0.890	1.188 (1.001–1.410)	0.048
Biliary disease	1.207 (0.766–1.900)	0.420	1.041 (0.786–1.379)	0.780	0.960 (0.724–1.272)	0.780

ALD, alcohol-related liver disease; HCV, hepatitis C virus infection; MASH, metabolic dysfunction-associated steatohepatitis; BMI, body mass index; MELD, Model for End-Stage Liver Disease; SLKT, simultaneous liver-kidney transplantation.

### Change of Waitlist Outcomes Before and After Allocation Policy Change

**FIGURE 3:**
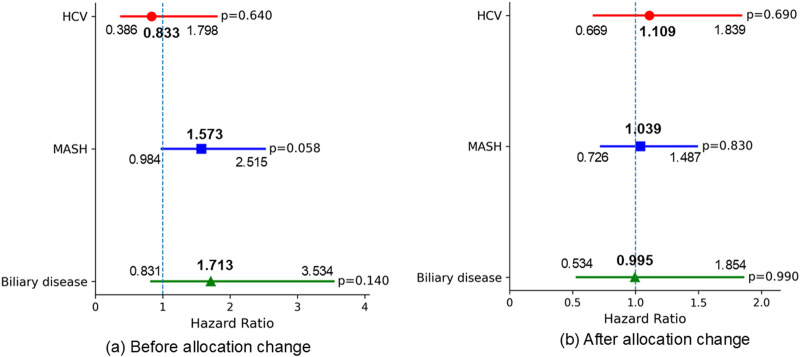
Multivariable Fine-Gray proportional hazards regression for 90-day waitlist mortality (reference: ALD). Adjusted for baseline characteristics at listing, including age, ascites, BMI, diabetes, encephalopathy, dialysis, life support measures, sex, MELD score, frailty, and race. **(a)** No significant differences in 90-day waitlist mortality were observed among the liver etiologies before the allocation policy change. **(b)** No significant differences in 90-day waitlist mortality were observed among the liver etiologies after the allocation policy change. ALD, alcohol-related liver disease; HCV, hepatitis C virus infection; MASH, metabolic dysfunction-associated steatohepatitis; BMI, body mass index; MELD, Model for End-Stage Liver Disease.

**FIGURE 4:**
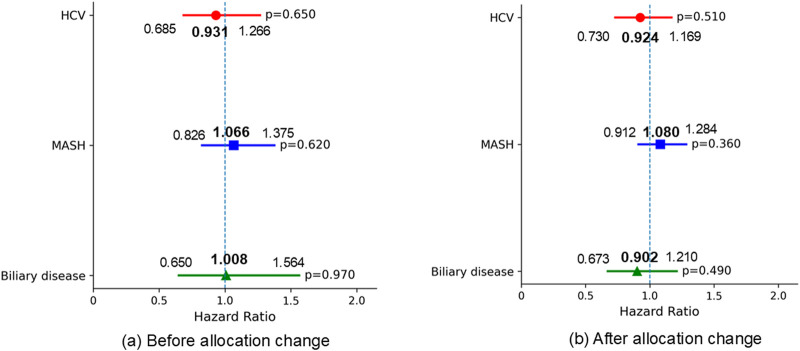
Multivariable Fine-Gray proportional hazards regression for 90-day SLKT probability (reference: ALD). Adjusted for baseline characteristics at listing, including age, ascites, BMI, diabetes, encephalopathy, dialysis, life support measures, sex, MELD score, frailty, and race. **(a)** No significant differences in 90-day SLKT probability were observed among the liver etiologies before the allocation policy change. **(b)** No significant differences in 90-day SLKT probability were observed among the liver etiologies after the allocation policy change. ALD, alcohol-related liver disease; HCV, hepatitis C virus infection; MASH, metabolic dysfunction-associated steatohepatitis; BMI, body mass index; MELD, Model for End-Stage Liver Disease.

To assess differences in waitlist outcomes among liver disease etiologies before and after the February 4, 2020 allocation policy change, we compared these outcomes across etiologies within each period. There were no significant differences in 90-day waitlist mortality across liver etiologies before the allocation policy change (Figure 3). There were no significant differences in 90-day SLKT probability across liver etiologies in both periods (Figure 4).

**FIGURE 5:**
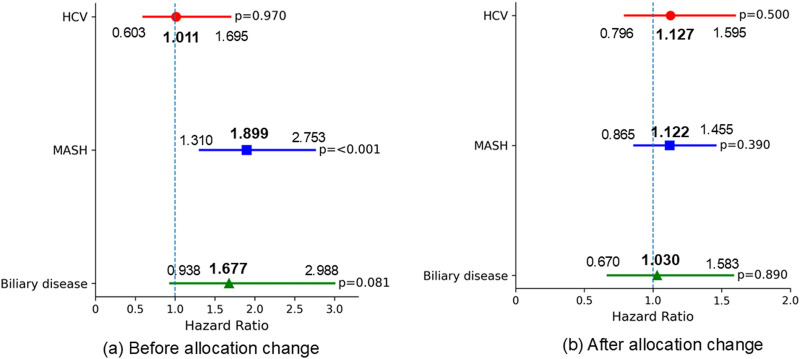
Multivariable Fine-Gray proportional hazards regression for 1-year waitlist mortality (reference: ALD). Adjusted for baseline characteristics at listing, including age, ascites, BMI, diabetes, encephalopathy, dialysis, life support measures, sex, MELD score, frailty, and race. **(a)** Patients with MASH had significantly worse 1-year waitlist mortality before the allocation policy change, compared to those with ALD. **(b)** No significant differences in 1-year waitlist mortality were observed among the liver etiologies after the allocation policy change. ALD, alcohol-related liver disease; HCV, hepatitis C virus infection; MASH, metabolic dysfunction-associated steatohepatitis; BMI, body mass index; MELD, Model for End-Stage Liver Disease.

**FIGURE 6:**
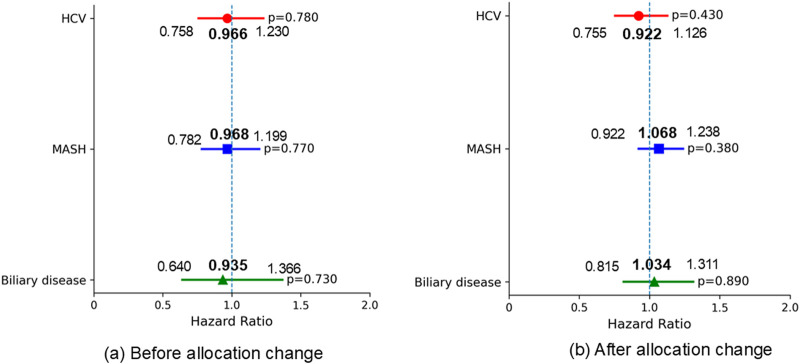
Multivariable Fine-Gray proportional hazards regression for 1-year SLKT probability (reference: ALD). Adjusted for baseline characteristics at listing, including age, ascites, BMI, diabetes, encephalopathy, dialysis, life support measures, sex, MELD score, frailty, and race. **(a)** No significant differences in 1-year SLKT probability were observed among the liver etiologies before the allocation policy change. **(b)** No significant differences in 1-year SLKT probability were observed among the liver etiologies after the allocation policy change. ALD, alcohol-related liver disease; HCV, hepatitis C virus infection; MASH, metabolic dysfunction-associated steatohepatitis; BMI, body mass index; MELD, Model for End-Stage Liver Disease.

Regarding 1-year waitlist mortality, patients with MASH had significantly worse mortality prior to the allocation policy change (HR 1.899, 95% CI 1.310–2.753, p < 0.001); however, this difference was no longer significant in the post-policy era. (HR 1.122, 95% CI 0.865–1.455, p = 0.390) (Figure 5) The 1-year SLKT probability in patients with MASH was comparable to that in patients with ALD in both periods (Figure 6).

### The Probability of Patient Death Among Patients Who Received SLKT

Among patients who underwent SLKT, those with MASH or biliary disease had a significantly higher risk of patient death at 1 year and 3 years post-transplantation compared with those with ALD, after adjustment for clinical factors at listing (Table 6). There was no significant difference in distribution of causes of death across liver etiologies (p = 0.858). Cardiovascular disease (CVD) and infection were the leading causes of death in all groups (Supplementary Table S2). In cause-specific analyses, patients with MASH or biliary disease were significantly associated with a higher risk of CVD-related death (Supplementary Table S3).

**TABLE 6:** Multivariable logistic regression for patient death among patients who received SLKT. Adjusted for baseline characteristics at listing, including age, ascites, BMI, diabetes, encephalopathy, dialysis, life support measures, frailty, sex, MELD score, and race.

Variables	Patient death within 1 year		Patient death within 3 years	
	HR (95% CI)	*p*-value	HR (95% CI)	*p*-value
Etiology of liver disease (ref; ALD)				
HCV	1.432 (0.919–2.231)	0.113	1.362 (0.963–1.927)	0.081
MASH	1.696 (1.199–2.398)	0.003	1.604 (1.226–2.098)	<0.001
Biliary disease	2.035 (1.192–3.472)	0.009	2.259 (1.498–3.406)	0.001

ALD, alcohol-related liver disease; HCV, hepatitis C virus infection; MASH, metabolic dysfunction-associated steatohepatitis; BMI, body mass index; MELD, Model for End-Stage Liver Disease.

## Discussion

In this study, we evaluated waitlist outcome variations across major liver disease etiologies, including ALD, HCV, MASH, and biliary diseases in SLKT. Waitlist outcomes varied significantly depending on the liver etiology in SLKT candidates. The Fine-Gray model revealed that patients with MASH had worse 1-year mortality compared to patients with ALD, after adjustment for patient characteristics at listing, whereas the 1-year SLKT probability was comparable between patients with MASH and patients with ALD. When patients were categorized by MELD score at listing (6–20, 21–29, and ≥30), in the middle MELD score group, patients with MASH had a significantly higher 1-year mortality compared to those with ALD. In addition, prior to the allocation policy change in 2020, patients with MASH experienced significantly higher 1-year waitlist mortality compared to those with ALD; however, this disparity was not observed following the policy change. During the post-transplantation period, recipients with MASH had worse 1-year and 3-year patient survival compared to those with ALD.

Prior studies have reported higher LT waitlist mortality in patients with MASH [6, 7, 18], which are consistent with our findings. MASH, previously known as non-alcoholic fatty liver disease, affects an estimated 30% of the global population, although prevalence varies considerably based on individual risk profiles [19]. MASH carries a risk of progression to advanced fibrosis and cirrhosis, and is associated with increased likelihood of HCC, LT, and liver-related mortality [20]. The impact of MASH extends beyond hepatic involvement, such as CVD, CKD, and type 2 diabetes mellitus [21]. Indeed, MASH has been related to an increased risk of CKD, with a recent meta-analysis indicating nearly a twofold higher risk of CKD among patients with MASH [22]. In addition, the majority of deaths among patients with MASH are attributable to CVD [21]. Thus, MASH is a clinically significant condition with systemic involvement, contributing to multi-organ dysfunction and increased mortality. In our study, post-SLKT mortality was higher in recipients with MASH compared with those with ALD. In cause-specific analyses, patients with MASH were associated with a higher risk of CVD-related death than those with ALD, suggesting that CVD risk may have contributed to the increased mortality in this group.

In contrast, several previous reports have suggested that MASH was not independently associated with increased mortality among LT waitlist candidates. Thuluvath et al reported that adjusted cumulative incidence of death on LT waitlist was comparable between MASH and autoimmune hepatitis cirrhosis [23]. However, dialysis and serum creatinine were identified as negative predictors of receiving LT. Thus, the renal dysfunction may play a vital role, particularly among candidates for SLKT. Wong et al analyzed waitlist outcomes among LT alone by disease etiology among patients registered in the United States between 2004 and 2013 [18]. In their report, patients with HCV were associated with higher 1-year waitlist mortality, compared to those with MASH. It should be noted that their study period ended in 2013, prior to the widespread availability of direct-acting antivirals (DAA) for HCV [24]. With the introduction of DAAs as a curative therapy, LT in patients with HCV now shows improved GS compared to the pre-DAA era [25]. This reflects a key difference in the historical context between that study and our current analysis, as our analysis was conducted during the DAA era.

In our study, ALD patients demonstrated the highest 90-day and 1-year LT alone (without receiving KT) probability. When adjusted for clinical factors at listing, this difference was no longer significant among liver etiologies. The reasons for not receiving KT were not clearly identifiable, whether due to renal recovery or the decision of dialysis. Thus, it was difficult to determine which etiology was more likely to be associated with kidney recovery and avoidance of SLKT. However, patients with ALD may have a higher potential for renal recovery than those with other etiologies. Meanwhile, according to the report by Singal et al, OPTN database analysis confirms the rising use of SLKT, particularly among patients with MASH cirrhosis [26]. Notably, the study highlights that SLKT has increased most rapidly in this group and that patients with MASH have worse renal outcomes compared to those with cholestatic or ALD. Future research is warranted to clarify whether the course of renal function differs by etiology among SLKT candidates.

Then, how should our findings be applied to optimize clinical management of SLKT candidates? When evaluating outcomes according to MELD score categories at listing, among SLKT candidates in the middle MELD subgroup, patients with MASH had higher 1-year waitlist mortality than those with ALD, while SLKT probability was comparable. Earlier referral for SLKT and more intensive management during the waiting period may be beneficial for patients with MASH in the middle MELD group. Although incorporation of liver etiology into the MELD score is challenging because overlap exists among etiologies, risk stratification cannot be fully achieved using the MELD score alone. MELD score-based assessment for urgency may be complemented by individual additional risk evaluation, careful follow-up while on the waitlist, including comorbidities, disease trajectory, and the likelihood of renal recovery. A strategy incorporating delayed KT and the safety-net may also be considered for patients with potential for renal recovery.

Since the adoption of the MELD score for organ allocation in 2002, the system of liver allocation and distribution has been changing dynamically with the goal of decreasing waitlist mortality and minimizing geographic variability in median MELD score at the time of transplantation, without compromising graft or patient survival [27]. We previously evaluated the impact of the MELD score, introduced in January 2016 for LT allocation, on waitlist and post-transplantation outcomes, using OPTN data [28]. The introduction of the MELD score improved LT waitlist outcomes by reducing mortality and increasing transplantation rates. In July 2023, MELD 3.0 was implemented to address sex-based disparities by incorporating additional variables such as serum albumin and female sex.^1^ According to retrospective analysis of MELD 3.0 reclassification using OPTN data, female SLKT candidates were more likely to have higher MELD 3.0 scores than their listing MELD/MELD-Na score, suggesting potential benefits for female candidates [29]. Other than the introduction of MELD score, changes to the system of liver allocation and distribution, including Regional Share 15, Regional Share for Status 1, Regional Share 35/National Share 15, and, most recently, the Acuity Circles Distribution Model, have been made [30–32]. On February 4, 2020, the United States implemented a new liver allocation policy, replacing Donation Service Areas (DSAs) with acuity circles as the primary geographic unit for organ distribution, shifting from a primarily local to a broader sharing system [33]. After this allocation policy change, transplantation volume increased nationwide and increased for both high MELD and low MELD centers [8]. Regarding the effect of the acuity circle policy on SLKT, Okumura et al demonstrated that, following the policy change, SLKT candidates had higher MELD scores and were more likely to receive SLKT, with no increase in 90-day waitlist mortality [34]. Additionally, post-transplantation outcomes including one-year patient, liver, and kidney graft survival were comparable between the pre- and post-AC periods, suggesting that the AC policy improved access to SLKT without compromising outcomes [34]. In our analysis, the differences in both 90-day and 1-year waitlist mortality by etiology were no longer observed after the 2020 allocation policy change. This finding suggests a temporal association between the 2020 allocation policy revision and mortality differences by etiology. This observation may also be influenced by the increasing use of marginal livers, including older donors or DCD (donation after circulatory death) [35], as well as growing use of DCD donors and machine perfusion in SLKT [36].

There are several limitations in our study using OPTN database. First, the etiology in this study was based solely on classifications within OPTN database, and cases with overlapping liver diseases could not be fully identified. In OPTN dataset, only 101 cases were identified as having both ALD and HCV during the study period. Given the limited sample size, this group was excluded in this analysis. Second, due to the retrospective cohort, a direct causal relationship with the acuity circle policy could not be determined. Of note, this study focused on patients without exception scores. The number of donors has been increasing consistently since 2010, reaching 14,905 in 2022, which represents a 7.5% increase compared with the previous year [37]. The increase in donor numbers may also have contributed to reducing disparities in mortality across different liver etiologies. Third, given the lack of comprehensive longitudinal data on serum creatinine and GFR prior to listing and throughout the waitlist period, precise classification of renal phenotypes (CKD or sustained AKI) was not available and thus could not be included in the analysis. Dialysis duration was also not included in the analysis due to a substantial amount of missing data. Fourth, there may be potential confounding by era effects such as the COVID-19 period, donor supply shifts and the MELD 3.0 implementation. However, it is difficult to precisely define discrete time periods for factors such as the COVID-19 era and shifts in donor supply. MELD 3.0 was implemented on July 13, 2023 and this study period was from January 2018 to March 2024. As the post-implementation period was relatively short (approximately 8 months), it was also difficult to evaluate its impact adequately. Finally, OPTN database lacks certain detailed clinical information, and unmeasured confounders may have influenced waitlist outcomes.

In conclusion, waitlist outcomes varied significantly depending on the etiology in SLKT. One-year waitlist mortality was higher in patients with MASH compared to those with ALD, despite similar SLKT probabilities. When stratified by MELD score at listing, patients with MASH in the middle score group had significantly higher mortality compared to those with ALD. The revised 2020 allocation policy may be temporally associated with changes in mortality disparities across different liver etiologies; however, further approaches to risk assessment tailored to the etiology of liver disease in SLKT are needed to ensure greater equity.

## Data Availability

The data reported here have been supplied by the UNOS as the contractor for the OPTN. The interpretation and reporting of these data are the responsibility of the author(s) and in no way should be seen as an official policy of or interpretation by the OPTN or the U.S. Government. This data can be found here: https://unos.org/data/.

## Footnotes

1
https://www.hrsa.gov/optn/data/allocation-calculators/meld-calculator
