# Supplementary figures and images for: Exploring Disease-Specific Waitlist Outcomes in Simultaneous Liver-Kidney Transplantation
Source: Transpl Int. 2026 Apr 13;39:16153. doi: 10.3389/ti.2026.16153 (PMC13111173; doi:10.3389/ti.2026.16153)

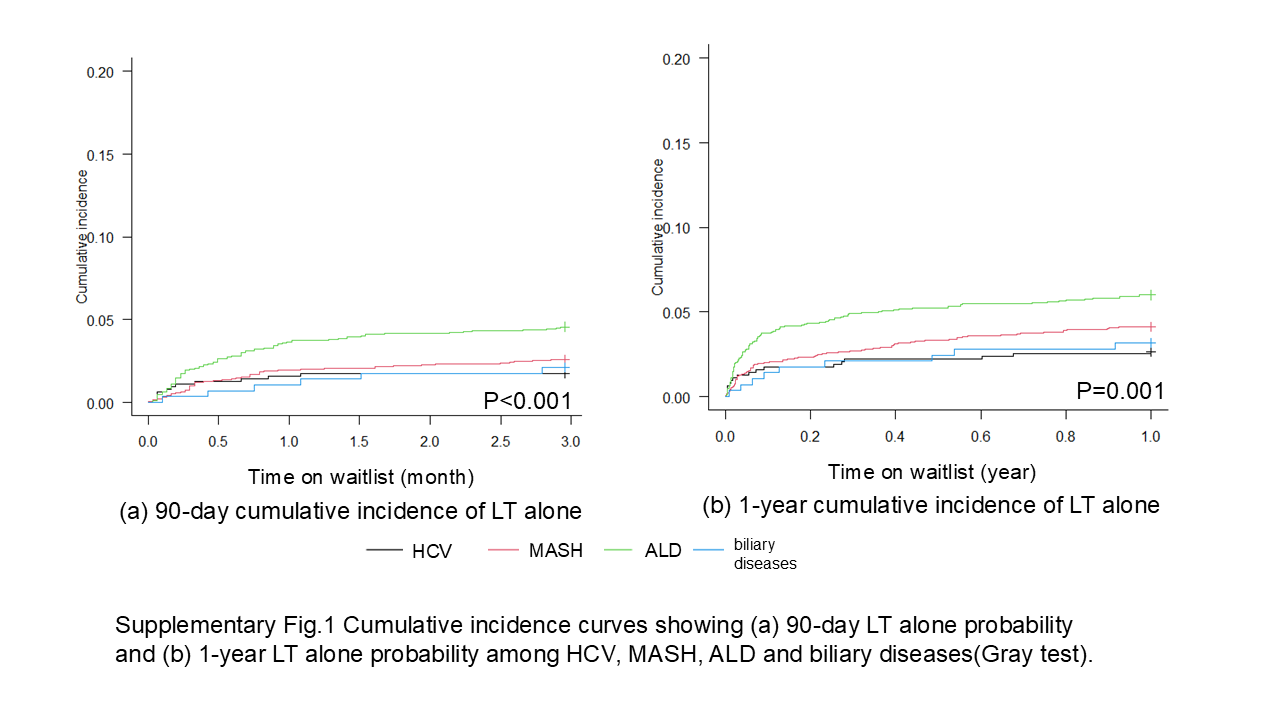

Supplement: Supplementary file 1 [file Image1.tif]
