# Supplementary material for: Exploring Disease-Specific Waitlist Outcomes in Simultaneous Liver-Kidney Transplantation
Source: Transpl Int. 2026 Apr 13;39:16153. doi: 10.3389/ti.2026.16153 (PMC13111173; doi:10.3389/ti.2026.16153)
Supplement: Supplementary file 2 [file DataSheet1.pdf]

Supplementary Table1. Multivariable Fine-Gray proportional hazards regression for the probability of LT alone (not undergoing KT due to recovery of kidney function or other reasons).

	90-day outcome		1-year outcome	
Variables	HR (95% CI)	*p*-value	HR (95% CI)	*p*-value
Age	0.999 (0.983-1.017)	0.990	0.997 (0.982-1.012)	0.640
Female	1.461 (1.046-2.042)	0.026	1.518 (1.152-2.001)	0.003
BMI	0.994 (0.966-1.023)	0.680	0.999 (0.976-1.023)	0.950
Diabetes	0.904 (0.599-1.364)	0.630	0.924 (0.669-1.277)	0.630
Etiology of liver disease (ref; ALD)				
HCV	0.672 (0.335-1.348)	0.260	0.611 (0.349-1.069)	0.084
MASH	0.719 (0.444-1.164)	0.180	0.742 (0.505-1.091)	0.130
Biliary disease	0.540 (0.229-1.277)	0.160	0.540 (0.266-1.095)	0.090
Race (ref: White)				
Black	0.943 (0.475-1.873)	0.870	0.951 (0.532-1.701)	0.870
Hispanic	1.709 (1.204-2.428)	0.003	1.519 (1.124-2.052)	<0.001
Asian	0.754 (0.237-2.399)	0.630	0.816 (0.332-2.007)	0.660
Others	0.274 (0.038-1.978)	0.200	0.389 (0.095-1.592)	0.190
MELD score	1.117 (1.090-1.143)	<0.001	1.068 (1.045-1.091)	<0.001
Dialysis	0.599 (0.434-0.828)	0.002	0.622 (0.474-0.816)	<0.001
Life support measures	0.866 (0.497-1.510)	0.610	0.988 (0.598-1.632)	0.960
Ascites	0.856 (0.601-1.219)	0.390	0.926 (0.693-1.237)	0.600
Encephalopathy(ref; none or mild)				
Severe	0.937 (0.570-1.540)	0.800	0.991 (0.645-1.523)	0.970
Frailty (ref; normal)				
Severe	0.894 (0.487-1.638)	0.720	0.739 (0.461-1.183)	0.210

ALD, alcohol-related liver disease; HCV, hepatitis C virus infection; MASH, metabolic dysfunction-associated steatohepatitis; BMI, body mass index: MELD, Model for End-Stage Liver Disease; SLKT, simultaneous liver-kidney transplantation

Supplementary Table2. Causes of death among SLKT recipients.

	ALD (N=72)	HCV (N=44)	MASH (N=141)	Biliary disease (N=15)
CVD (%)	18 (25.0)	14 (31.8)	48 (34.0)	6 (40.0)
Infection (%)	29 (40.3)	14 (31.8)	52 (36.9)	6 (40.0)
Malignancy (%)	11 (15.3)	6 (13.6)	20 (14.2)	1 (6.7)

*P=0.858

ALD, alcohol-related liver disease; HCV, hepatitis C virus infection; MASH, metabolic dysfunction-associated steatohepatitis; CVD, cardio vascular disease

Supplementary Table3. Multivariable Fine-Gray proportional hazards regression for cause-specific mortality. Adjusted for baseline characteristics at listing, including age, ascites, BMI, diabetes, encephalopathy, dialysis, life support measures, frailty, sex, MELD score, and race.

(a) Death by CVD

	Patient death within 1 year		Patient death within 3 years	
Variables	HR (95% CI)	*p*-value	HR (95% CI)	*p*-value
Etiology of liver disease (ref; ALD)				
HCV	1.365 (0.788-2.364)	0.270	1.290 (0.829-2.008)	0.260
MASH	1.603 (1.007-2.551)	0.046	1.675 (1.173-2.392)	0.005
Biliary disease	2.187 (1.136-4.209)	0.019	2.602 (1.574-4.301)	<0.001

(b) Death by infection

	Patient death within 1 year		Patient death within 3 years	
Variables	HR (95% CI)	*p*-value	HR (95% CI)	*p*-value
Etiology of liver disease (ref; ALD)				
HCV	1.174 (0.413-3.337)	0.760	0.910 (0.387-2.140)	0.830
MASH	1.382 (0.642-2.975)	0.410	1.128 (0.639-1.993)	0.680
Biliary disease	1.643 (0.510-5.295)	0.410	1.353 (0.521-3.512)	0.530

(c) Death by malignancy

	Patient death within 1 year		Patient death within 3 years	
Variables	HR (95% CI)	*p*-value	HR (95% CI)	*p*-value
Etiology of liver disease (ref; ALD)				
HCV	1.386 (0.135-14.29)	0.780	2.156 (0.676-6.874)	0.190
MASH	3.264 (0.349-30.54)	0.300	1.687 (0.696-4.090)	0.250
Biliary disease	Not calculable		1.681 (0.213-13.25)	0.620

ALD, alcohol-related liver disease; HCV, hepatitis C virus infection; MASH, metabolic dysfunction-associated steatohepatitis; CVD, cardio vascular disease
